# PARP inhibitors in gastric cancer: beacon of hope

**DOI:** 10.1186/s13046-021-02005-6

**Published:** 2021-06-24

**Authors:** Yali Wang, Kun Zheng, Yongbiao Huang, Hua Xiong, Jinfang Su, Rui Chen, Yanmei Zou

**Affiliations:** Department of Oncology, Tongji Hospital, Tongji Medical College, Huazhong University of Science and Technology, Jiefang Road, Wuhan, 430030 Hubei China

**Keywords:** PARP inhibitor, Gastric cancer, DNA damage response, Cancer treatment

## Abstract

Defects in the DNA damage response (DDR) can lead to genome instability, producing mutations or aberrations that promote the development and progression of cancer. But it also confers such cells vulnerable to cell death when they inhibit DNA damage repair. Poly (ADP-ribose) polymerase (PARP) plays a central role in many cellular processes, including DNA repair, replication, and transcription. PARP induces the occurrence of poly (ADP-ribosylation) (PARylation) when DNA single strand breaks (SSB) occur. PARP and various proteins can interact directly or indirectly through PARylation to regulate DNA repair. Inhibitors that directly target PARP have been found to block the SSB repair pathway, triggering homologous recombination deficiency (HRD) cancers to form synthetic lethal concepts that represent an anticancer strategy. It has therefore been investigated in many cancer types for more effective anti-cancer strategies, including gastric cancer (GC). This review describes the antitumor mechanisms of PARP inhibitors (PARPis), and the preclinical and clinical progress of PARPis as monotherapy and combination therapy in GC.

## Background

Gastric cancer (GC) is one of the most common cancers worldwide and the second leading cause of cancer-related deaths [1]. Due to its high incidence, high mortality rate, and extremely poor prognosis, it poses a serious threat to human health and life. Currently, surgical resection is the best treatment for patients with early GC [2], and chemotherapy is the most important treatment for patients who cannot undergo surgical resection or have advanced metastases [3]. However, GC is biologically and genetically highly heterogeneous [4], resulting in less than optimal results from surgical resection and chemotherapy. It is therefore urgent to explore more effective therapeutic strategies. The idea that DNA damage-deficient mechanisms promote tumorigenesis was recognised 20 years ago [5], and direct or indirect targeting of DNA damage repair pathways had emerged in cancer treatment approaches, and the clinical success of PARP inhibitors (PARPis) had made “synthetic lethal” anti-cancer therapies a new hope.

The replication fork encounters a lot of DNA damage during each cell cycle, and cells have developed several specific pathways to prevent specific DNA damage from causing cell “survival” problems [5, 6]. Among them, poly (ADP-ribose) polymerase (PARP) plays an irreplaceable role in the repair of DNA single-strand breaks (SSB) [7]. One of the most dramatic post-translational modifications caused by DNA damage is protein poly (ADP-ribosylation) (PARylation), catalyzed by members of the PARP nicotinamide adenine dinucleotide (NAD+) dependent ADP-ribosyltransferase superfamily [8], which is used to accelerate damage repair and plays a key role in cell fate determination. When SSB are not repaired after PARPis, it is transformed into DNA double Strand Breaks (DSB) in the S phase. DSB is considered one of the most lethal forms of DNA damage [9]. In normal cells, DSB can be repaired by homologous recombination repair (HRR). However, in cancers with homologous recombination deficiency (HRD), DSB can only be repaired with low-fidelity forms of DNA repair (e.g. Non-homologous end joining, NHEJ) after the use of PARPis, which will lead to a significant increase in genomic instability and cell death due to apoptosis or mitotic catastrophe [10]. This “synthetic lethality” occurs when there is a strong lethal synergy between two non-lethal events [11].

HRD related genotypes have been found in GC for many years [12]. To date, PARPis have been used as “gold finger” drugs in various cancer therapies, which has made them a focus of GC research. Nowadays, the efficacy of PARPis has been validated in various clinical trials in GC. Therefore, this review will first briefly describe the antitumor mechanisms of PARPis and then outline the preclinical and clinical progress of PARPis as monotherapy and combination therapy in GC.

## The mechanism of PARPis

### ADP-ribosylation

One of the most common tools to induce a rapid change in the cellular environment is the post-translational modification (PTM) of proteins by addition of chemical moieties, such as phosphate, acyl (most commonly methyl and acetate), small proteins or sugars [13]. Of these, the least studied of the various histone modifications is likely to be ADP-ribosylation. ADP-ribosylation also is a PTM, in which ADP-ribosyltransferases use NAD+ to modify target proteins with ADP-ribose. This modification, occurring as mono- or poly-ADP-ribosylation [14], can alter the physical and chemical properties of target proteins [15]. ADP-ribosylation is associated with many cellular processes, including different forms of stress and metabolism, such as DNA repair, transcription, regulation of meristem function, telomere length and senescence, protein degradation, apoptosis and necrosis [16–18]. This may be due to the fact that poly (ADP-ribosylation) is usually associated with the relaxation of chromatin structure in target proteins.

**Fig. 1:**
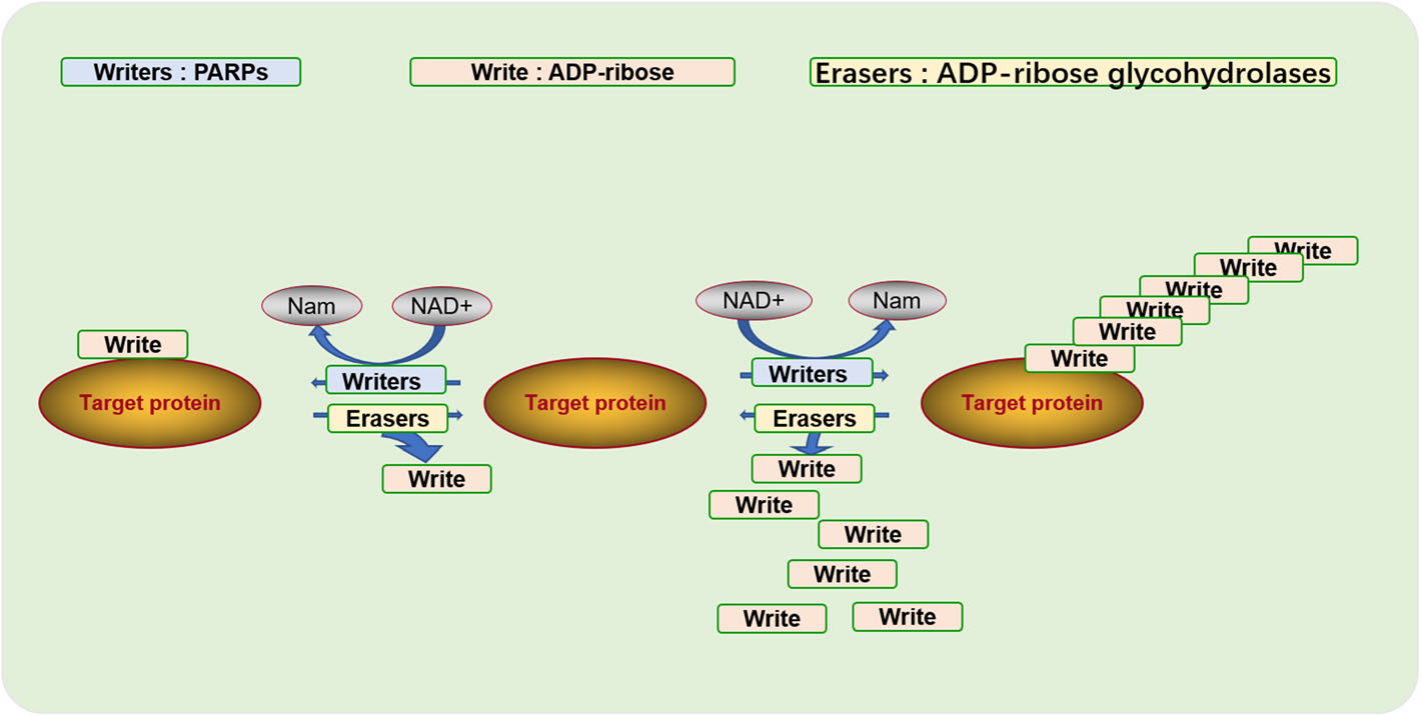
Regulation of reversible ADP-ribosylation. ADP-ribosylation is a post-translational modification of the protein, the use of NAD+ as a donor, PARPs act as synthases to ligate ADP-ribose to target proteins, ADP-ribose is also a reversible process, ADP-ribose can be removed by hydrolytic enzymes, such as PARG, ARH3 and so on

The enzymes involved in ADP-ribosylation are mainly those of the ART (ADP-ribosyltransferase) superfamily, characterized by structural homology of the ART domain to either diphtheria toxin (ART diphtheria toxin like, ARTD) or cholera toxin (ART cholera toxin like, ARTC). In mammals, the only known proteins that can be poly (ADP-ribosyl) ed. are members of the PARP family [19]. Thus, PARPs can be considered as ADP-ribose “writers” (writers being the enzymes that catalyse the formation of ADP-ribose modifications), which attach (“write”) ADP-ribose units to substrate proteins in a covalent manner. Removal of the ADP-ribose chain is catalyzed by “erasers” including PAR glycolytic enzymes (PARG), ADP-ribosyl hydrolase 3 (ARH3), TARG and MacroD1/D2 [20, 21] (Fig. 1).

### The family of PARP

The PARP protein family consists of 17 members that have distinct structural domains, activities, subcellular localizations, and functions [22, 23]. However, they share a conserved catalytic PARP domain fold that is homologous to the diphtheria toxin ART fold and is therefore also known as diphtheria toxin-like ADP-ribosyltransferase (ARTDs) [8, 24]. Because poly (ADP-ribosyl) isomerization is often associated with the relaxation of chromatin structure, PARP may play a central role in many cellular processes, including DNA repair, replication, and transcription. Based on their structural domains and functions, the different PARPs can be broadly classified as DNA-dependent PARPs (PARP1, PARP2, and PARP3), Tankyrases (PARP5a and PARP5b), Cys-Cys-Cys-His zinc finger (CCCH)-containing and WWE PAR-binding domain-containing PARPs (PARP7, PARP12, PARP13.1, and PARP13.2), and PAR-binding macrodomain-containing “macro” PARPs (PARP9, PARP14, and PARP15). The remaining PARPs are referred to as unclassified PARPs [20, 23] (Table 1).

**Table 1:** Members of the PARP family

Subfamily	Family members	alternative names	catalytic activities
DNA-dependent PARPs	PARP1	ARTD1	Poly
	PARP2	ARTD2	Poly
	PARP3	ARTD3	Mono
Tankyrases	PARP5a	ARTD5/tankyrase1	Poly
	PARP5b	ARTD6/tankyrase1	Poly
(CCCH) Zn finger/WWE	PARP7	ARTD14/TiPARP	Mono
	PARP12	ARTD12/ZC3HDC1	Mono
	PARP13	ARTD13/ZAP	Inactive
Marco	PARP9	ARTD9/BAL1	Mono
	PARP14	ARTD8/BAL2	Mono
	PARP15	ARTD7/BAL3	Mono
Unclassified	PARP4	ARTD4/vPARP	Mono
	PARP6	ARTD17	Mono
	PARP8	ARTD16	NA
	PARP10	ARTD10	Mono
	PARP11	ARTD11	Mono
	PARP16	ARTD15	Mono

PARP involvement in the cellular response to DNA damage has long been appreciated and continues to actively develop [25, 26]. PARP, a sensor of DNA damage, coordinates DNA repair processes by providing poly-ADP-ribose chains that function as interaction platforms for repair and signaling proteins [14]. The extent of the PARylation response to DNA damage largely depends on the nature and amount of DNA breaks produced. In response to low levels of DNA lesions, PARP activity favors repair and survival. In the presence of extensive DNA injury as observed during ischemia/reperfusion and inflammatory conditions, the massive production of PAR ultimately causes cell-death via at least two distinct mechanisms: energy-failure induced necrosis or apoptosis-inducing factor (AIF) dependent apoptosis [27]. A tight regulation of PARylation homeostasis is therefore of critical importance for efficient repair when cells are exposed to sub-lethal doses of DNA damage [28]. PARP activation consumes NAD+ and alters AMP:ATP ratios through the actions of PARG, which hydrolyses pADPr into ADP-ribose, and of the ADP-ribose pyrophosphohydrolase NUDIX enzymes, which cleave ADP-ribose into AMP and phospho-ribose [29].

### PARP in cancer

Defective DNA repair is a common hallmark of cancer [30]. Compared with normal cells, cancer DDR is characterized by losing one or more DDR pathway or capability during their generation, leading to a greater dependency on the remaining pathways [31, 32]. The DDR is a cellular response to exogenous and endogenous genotoxic damage that may result in SSB and DSB. Of these, DSB are considered one of the most lethal forms of DNA damage [33]. In normal cells, the accumulations of DSB are repaired preferentially by HR rather than NHEJ [34, 35]. HR is a high-fidelity repair pathway that utilizes the sister copy of the damaged DNA as a template, leading to the reconstitution of the original sequence [36, 37]. In contrast, NHEJ is intrinsically error-prone, modifies the broken DNA ends, and ligates them together with little or no homology, generating deletions or insertions [35]. Tumor cells, lacking a functional HR pathway will be more dependent on SSB repair mechanisms. PARP itself constitutes a component of the base excision repair (BER) pathway [38]. It is well established that PARP proteins bind to SSB and that poly-ADP-ribose polymerase, as a cofactor, is required for the effective repair of SSB [39]. It accomplishes SSB repair by recruiting XRCC1 and ligase III to the site of injury [40–42]. Meanwhile, PARP contributes to DSB repair as a component of an alternative pathway of NHEJ [43]. PARP1 also contributes to HR system functioning by recruiting critical DNA repair factors such as NBS1 and MRE11 to sites of DSB and by preventing Ku70/80 proteins from binding to areas of DNA damage. Ku proteins are essential components of the error-prone NHEJ pathway, and PARP1 exerts an active role in its inhibition [44, 45]. PARP3 has newly been acknowledged facilitating the process of NHEJ [46]. Thus, PARP can inhibit classical non-homologous end joining (cNHEJ) to some extent, allowing PARP to be highly activated in HRD tumor cells [47].

### “ Synthetic lethality “ of PARPis

Because different DNA-repair pathways can overlap in function, and as one pathway can sometimes ‘back-up’ for defects in another, inhibition of such pathways present in cancer cells could have a greater impact on the tumor tissues than normal tissues. Given the role of PARP in BER, inhibitors targeting PARP were developed in cancers with HRD to cause the accumulation of persistent SSB.

The main mechanism of PARPis involves the inhibition of PARP enzymatic activity (formation of poly ADP-ribose chains from NAD+), which is required for both relaxing chromatin and PARP dissociation from the DNA that occurs following auto-modification. Both of these events are required to facilitate SSB repair, and the structures of the PARPis are built around an NAD+ mimetic core. Consequently, competitive inhibition could substitute inactivated enzyme for NAD+ thus preventing NAD+ utilization on PARP protein, finally generating a potential block for cellular DNA replication [48]. The aforementioned process could trap PARP on the DNA, preventing auto-PARylation and PARP release from the site of the DNA lesion [49, 50]. Trapped PARP–DNA complexes were more cytotoxic than unrepaired SSB caused by PARP inactivation, arguing that PARPis act in part as poisons that trap PARP enzyme on DNA [50, 51]. At the same time, trapped PARP-DNA complexes can lead to the stalling and/or collapsing of replication forks, resulting in the generation of more deleterious DSB [52]. A second mechanism of PARP inhibitors demonstrates that PARP can inhibit cNHEJ [46]. As a result, PARPis will enhance NHEJ. NHEJ will result in a significant increase in genomic instability that over multiple rounds of replication will become unsustainable and result in tumor cell death [47]. Recently, a third mechanism for the sensitivity of PARPis has been discovered that PARPis decreases efficiency of mutagenic microhomology-mediated end joining (MMEJ)-based repair [53]. HR factors suppress MMEJ following DSB resection. Due to the defect in HR, tumor cells can rely on MMEJ (or Alt-EJ pathway) for the DSB repair pathway. This pathway is dependent on PARP and the trans-disease polymerase (POLQ). In fact, PARP is required for efficient recruitment of POLQ to DSB. Thus, inhibitors of PARP or POLQ will block the Alt-EJ pathway and kill HR-deficient tumor cells [53–55].

**Fig. 2:**
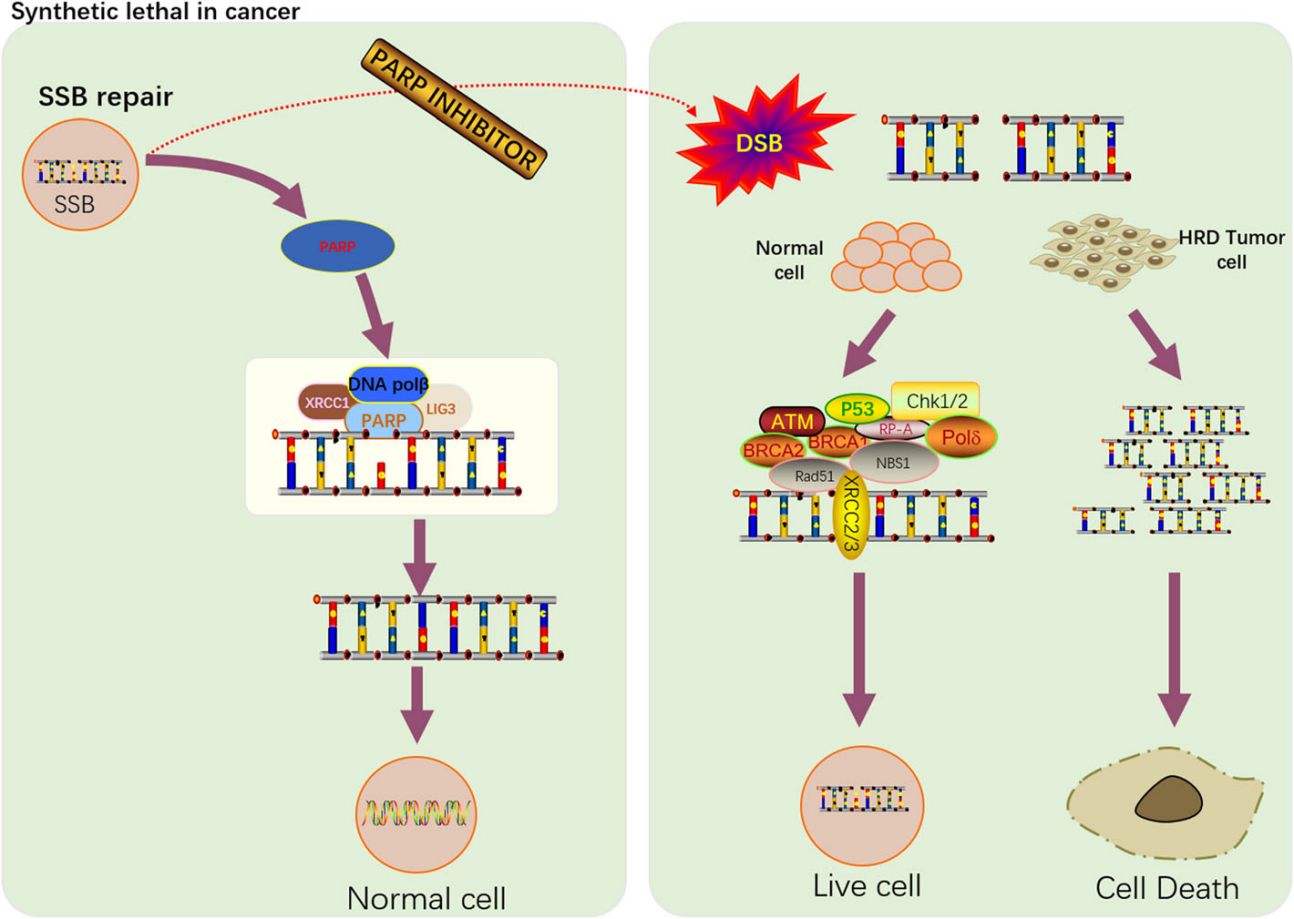
The principle of synthetic lethality. PARP is involved in SSB repair, and when PARP inhibitors can bind competitively with PARP, it leads to a large accumulation of SSB in the cell that cannot be repaired in time. Unrepaired SSB will lead to the collapse of replication fork, resulting in DSB. In normal cells, DSB can be repaired. In HRD tumor cells, DSB can not be repaired, resulting in a significant increase in the probability of tumor cell death. According to the synthetic lethal principle, PARP inhibitors can selectively kill tumor cells without affecting normal somatic cells

Although the precise mechanism by which PARPis kill tumor cells remains to be fully clarified, the anticancer effect is attributed to catalytic inhibition of PARP that block repair of SSB. While PARPis is well-tolerated by normal cells, this effect of PARPis is more likely observed in tumor cells with a HR- deficient background [48]. As a result of defective enzymatic function induced by PARPis, the accumulation of SSB is subsequently encountered by replication forks and generates potentially lethal DSB that need to be fixed [56]. In the absence of a functional HR pathway, highly specific PARPis induce DNA damage and tumor-restricted gene repair functions, resulting in the loss of required DNA repair functions and the death of tumor cells, leading to the concept of “synthetic lethality” [57, 58] (Fig. 2).

PARPis have demonstrated their clinical efficacy in HR-deficient cancer entities, for example, some of PARPis had been used clinically to treat breast cancers with BRCA 1 or 2 pure deletions [59, 60]. BRCA1 and BRCA2 are key components of the HR pathway. Initial observations were quickly expanded and it was demonstrated that defects in other HR components (e.g., RAD51, RPA1, ATM, ATR, CHK2, TOPBP1, ARID1A, etc.) were also associated with PARP inhibitor sensitivity [61–63]. Effect of PARPis may depend on the specific genetic environment of the specific tumor. For example, the survivals of BRCA1/2-deficient human breast tumors are dependent on the PARP/POLQ pathway, whereas the mechanisms corresponding to other mutations may be different [51].

There are some studies showing a survival benefit of PARPis in patients with tumors regardless of BRCA1/2 mutation status or HR repair status [64]. The findings indicate that PARPis treatment induces IFN-mediated antitumor immune responses. PARPis generate cytosolic dsDNA, which activate stimulator of IFN genes (STING) signaling and its associated transcription programs. These critical changes amplify STING signaling, and promote tumor-infiltrating lymphocytes (TIL) and antitumor immunity, which can be further enhanced through immune checkpoint blockade [65]. This makes us more aware of the deeper possibilities of the PARPis mechanism for better use of PARPis.

### Drugs targeting PARP

Since 2014, three PARPis — olaparib, rucaparib and niraparib—have been approved by the Food and Drug Administration (FDA) and European Medicine Agency (EMA) for application in ovarian cancer [66]. For instance, olaparib and rucaparib have been approved to treat BRCA-defective prostate cancer patients [67]. The FDA announced the approval of talazoparib for the treatment of harmful or suspected harmful germline BRCA-mutant, HER2-negative locally advanced or metastatic breast cancer. Studies involving other PARPis have also shown encouraging clinical results and are likely to receive additional approvals in the near future, such as veliparib, pamiparib (BGB-290), fluzoparib, E7449 and IMP4297. We have classified them into Pan-PARPs as well as Specific PARPs depending on the target (Table 2). The majority of the PARPis identified to date are pan-PARPis that target multiple PARPs, and the variety of PARP inhibitors is remarkable. We searched the term “PARPi” on Clinicaltrial.gov and found 298 relevant studies, of which 75 clinical trials have been completed as a single drug or in combination with other therapies (https:// clinicaltrials. Gov //). There are up to a dozen types of PARPi drugs in clinical trials; there are also up to a dozen drugs in preclinical studies (Table 2).

**Table 2:** A collection of preclinical as well as clinical PARPis drugs

Targeted	Drug name	Target	Study Phase	Reference
Pan-PARP	Olaparib (Lynparza)	PARP1, PARP2	FDA approval (OC)	[68]
	Niraparib (ZL-2306)	PARP1, PARP2	FDA approval (OC)	[69]
	Rucaparib (PF-01367338)	PARP	FDA approval (OC)	[70]
	Talazoparib (BMN-673)	PARP1, PARP2	FDA approval (BC)	[71]
	Veliparib (ABT-888)	PARP1, PARP2	phase III (Squamous Non-Small Cell Lung Cancer)	NCT02106546
	2X-121	TNK 1/2	phase II (Advanced Ovarian Cancer)	NCT03878849
	CEP-9722	PARP, TNK	phase I (Advanced Solid Tumors)	NCT04335604
	BGB-290 (Pamiparib)	PARP1, PARP2	phase II (Advanced or Inoperable Gastric Cancer)	NCT03427814
	JPI-547	PARP, TNK	phase I (Advanced Solid Tumors)	NCT04335604
	INO-1001	PARP	phase I (Melanoma)	NCT00272415
	A-966492	PARP1, PARP2	Preclinical	[72]
	PJ-34 HCI	PARP	Preclinical	[73]
	UPF1069	PARP1, PARP2	Preclinical	[74]
	RK-287107	TNK 1/2	Preclinical	[75]
	IMP4297	PARP1, PARP2	phase I (Advanced Solid Tumors and Small Cell Lung Cancer)	NCT04434482
	E7449	PARP1, PARP2	Phase I/II (Advanced Solid Tumors/B-cell Malignancies)	NCT01618136
	NU1025	PARP	Preclinical	[76]
	AZD2461	PARP1	phase I (Refractory Solid Tumors)	NCT01247168
	NMS-03305293	PARP1	phase I (Selected Advanced/Metastatic Solid Tumors)	NCT04182516
	Fluzoparib (SHR3162)	PARP1	phase II (Relapsed Ovarian Cancer)	NCT04517357
	3-aminobenzamide	PARP1	Preclinical	[77]
	AG-14361	PARP1	Preclinical	[78]
	NMS-P118	PARP1	Preclinical	[79]
	ME0328	PARP3	Preclinical	[80]
NA	IDX-1197	NA	phase II (HRR Mutated Solid Tumors)	NCT04174716
	E7016	NA	phase I (Advanced Solid Tumors)	NCT01127178

### PARPis in gastric Cancer

**Fig. 3:**
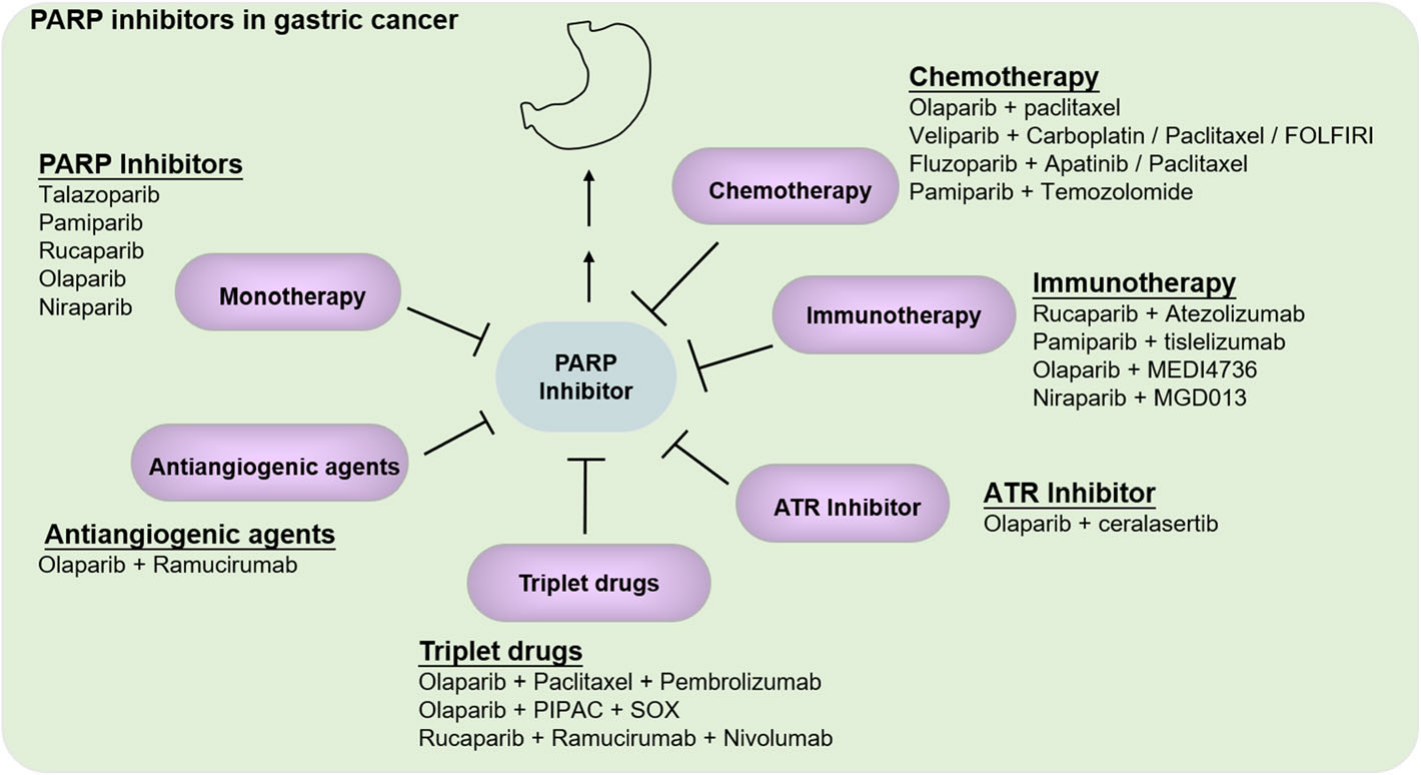
PARP inhibitors in gastric cancer. PARP inhibitors are used as single agents or combination therapy agents in the clinical or preclinical treatment of gastric cancer

GC is one of the most common malignant tumors of the digestive system [1], which is a highly heterogeneous disease. Although emerging targeted strategies and immunotherapies have brought new hope to antitumor therapy, options for advanced GC with high heterogeneity are still few. Only three drugs have been currently approved (trastuzumab, lamocirumab, and apatinib), but the prognosis for advanced GC remains poor. Hence, the novel strategies are urgently needed for advanced GC [81]. The most recurrently mutated genes of GC, which were validated in a separate cohort of 216 cases by targeted sequencing, were members of the homologous recombination DNA repair, WNT and PI3K-ERBB pathways [82]. Deficiencies in HR are extensively seen in gastric cancer. Mutations in PALB2, BRCA1, BRCA2, ARID1A, ATM or RAD51C genes, which regulate homologous DNA recombination, were widely identified in GC [49, 82, 83]. Tumors that developed in patients with these mutations had a mutation signature associated with somatic HRD. Hence, based on the above description of PARPis function, it is enough to see that the application of PARPis in GC is promising. Here we will list some studies about single PARPis drugs investigated in GC either in the preclinical or clinical phase as follows (Fig. 3).

### Monotherapy of PARPis

In the treatment of GC, PARPis has some non-DNA repair functions in addition to the above well-known functions of blocking DNA repair. Vascular endothelial growth factor (VEGF) and vascular endothelial growth factor receptor 2 (VEGFR2)-mediated signaling and angiogenesis can contribute to the pathogenesis and progression of GC [84]. PARPis cause loss of ERK2 stimulation by decreasing the activity of critical pro-angiogenic factors including VEGF, transmembrane signaling protein syndecan-4 (SDC-4) and hypoxia inducible factor (HIF). This ultimately results in reduced angiogenesis and inflammation [84–86]. Furthermore, inhibition of PARP1 is associated with transcriptional silencing through accumulation of DNA methylation and CpG island hypermethylation throughout the genome [87]. PARP1 can also hinder DNA methylation by dimerization with DNA (cytosine-5-)-methyltransferase 1 (DNMT1), a methyltransferase found overexpressed in gastrointestinal tract carcinomas, resulting in inhibition of its methyltransferase activity [87]. Recently, PARP1 inhibition has been addressed to attenuate the AKT-associated phosphorylation of forkhead box O (FOXO) transcription factors [88]. PARP1 is known to indirectly regulate FOXO3A via the NF-κB or AKT pathway and plays an important role in the progression of GC [89, 90].

### Preclinical

In preclinical studies, GC cell lines showed sensitivity to olaparib, especially those with low ataxia telangiectasia mutant (ATM) protein expression or RAD51C-Deficient [91, 92]. The possible cause is the deficiency of HR pathway due to the loss of these proteins.

### Clinical

Clinical trials about PARPis are ongoing. Olaparib was the first PARP inhibitor introduced in clinical practice [93]. A multicenter, non-randomized, phase II trial (NCT03829345) involving GC was recruiting, patients will be administered olaparib orally twice daily (BD) at 300 mg continuously for each 28 days cycle. Other single-drug phase II trials, including Talazoparib (NCT04550494), pamiparib (NCT03427814), Rucaparib (NCT04171700), Niraparib (NCT03840967) (Table 3), are also underway and the results are promising.

**Table 3:** Summary of clinical trials involving PARPis

Sensitization	Study Title	Status	Intervention		Most advanced clinical phase	Setting	Trial identifier
			PARPis	synergistic members			
–	Measuring the Effects of Talazoparib in Patients With Advanced Cancer and DNA Repair Variations	Not yet recruiting	Talazoparib	–	II	Clinical Stage III/ IV Gastric Cancer AJCC v8	NCT04550494
	Study of BGB-290 or Placebo in Participants With Advanced or Inoperable Gastric Cancer	Active, not recruiting	pamiparib	–	II	Advanced or Inoperable Gastric Cancer	NCT03427814
	A Study to Evaluate Rucaparib in Patients With Solid Tumors and With Deleterious Mutations in HRR Genes (LODESTAR)	Recruiting	Rucaparib	–	II	stomach cancer	NCT04171700
	A Translational Study of Single Agent Olaparib in the Treatment of Advanced Oesophagogastric Cancer (SOLAR)	Recruiting	Olaparib	–	II	Gastric and oesophageal cancers	NCT03829345
	A Study Evaluating Safety and Efficacy of Niraparib in Patients With Previously Treated Metastatic Esophageal/Gastroesophageal Junction/Proximal Gastric Adenocarcinoma	Recruiting	Niraparib	–	II	Gastric Cancer	NCT03840967
chemotherapy	Efficacy Study of Olaparib With Paclitaxel Versus Paclitaxel in Gastric Cancer Patients	Active, not recruiting	olaparib	paclitaxel	II	Recurrent or Metastatic Gastric Cancer Who Progress Following First-line Therapy	NCT01063517
	A Study Evaluating Veliparib as a Single Agent or in Combination With Chemotherapy in Subjects With Solid Tumors	Completed	Veliparib	Carboplatin, Paclitaxel, FOLFIRI	I	Gastric Cancer	NCT02033551
	A Study of Fluzoparib Given in Combination With Apatinib and Paclitaxel in Gastric Cancer Patients	NA	Fluzoparib	Apatinib, Paclitaxel	I	Recurrent and Metastatic Gastric Cancer	NCT03026881
	Evaluating the Safety and Tolerability of the Poly-ADP Ribose (PARP) Inhibitor With FOLFIRI in Subjects With Solid Tumor	Completed	Veliparib	FOLFIRI	I	Gastric Cancer	NCT01123876
	Efficacy and Safety Study of Olaparib in Combination With Paclitaxel to Treat Advanced Gastric Cancer	Active, not recruiting	Olaparib	Paclitaxel	III	Gastric Cancer	NCT01924533
	A Phase 1b Study to Assess the Safety, Tolerability and Clinical Activity of BGB-290 in Combination With Temozolomide (TMZ) in Subjects With Locally Advanced or Metastatic Solid Tumors	Recruiting	BGB-290	Temozolomide	I/II	Gastric Cancer	NCT03150810
antiangiogenic agents	Olaparib and Ramucirumab in Treating Patients With Metastatic or Locally Recurrent Gastric or Gastroesophageal Junction Cancer That Cannot Be Removed by Surgery	Recruiting	Olaparib	Ramucirumab	I/II	Metastatic or Locally Recurrent Gastric cancer	NCT03008278
ATR	Ascending Doses of Ceralasertib in Combination With Chemotherapy and/or Novel Anti Cancer Agents	Recruiting	Olaparib	ceralasertib	I/II	Gastric Cancer	NCT02264678
immunotherapy	Efficacy and Safety of the Combination of Rucaparib (PARP Inhibitor) and Atezolizumab (Anti-PD-L1 Antibody) in Patients With DNA Repair-deficient or Platinum-sensitive Solid Tumors (ARIANES)	Recruiting	Rucaparib	Atezolizumab	II	Gastric or gastro-esophageal junction adenocarcinoma	NCT04276376
	The Safety, Pharmacokinetics and Antitumor Activity of BGB-A317 in Combination With BGB-290 in Participants With Advanced Solid Tumors	Completed	BGB-290	tislelizumab	I/Ib	(HER2)-negative gastric or gastroesophageal junction cancer	NCT02660034
	A Phase I/II Study of MEDI4736 in Combination With Olaparib in Patients With Advanced Solid Tumors (MEDIOLA)	Active, not recruiting	Olaparib	MEDI4736	I/II	Metastatic or relapsed Gastric cancer (adenocarcinoma)	NCT02734004
	A Study of Niraparib Combined With MGD013 in Gastric/Gastroesophageal Junction Cancer	Recruiting	Niraparib	MGD013	I	Gastric Cancer	NCT04178460
triplet drugs	Paclitaxel, Pembrolizumab and Olaparib in Previously Treated Advanced Gastric Adenocarcinoma	Recruiting	Olaparib	Paclitaxel, Pembrolizumab	II	Advanced Gastric Adenocarcinoma	NCT04209686
	Neoadjuvant Chemotherapy With PISOXO for Locally-invaded-gastric Cancer (LIGC)	Not yet recruiting	Olaparib	PIPAC, SOX	I/II	Locally-invaded-gastric Cancer	NCT04410887
	Rucaparib Plus Ramucirumab With or Without Nivolumab in Advanced Gastric and Esophageal Adenocarcinoma (RiME)	Recruiting	Rucaparib	Ramucirumab, Nivolumab	I/II	Advanced Gastric and Esophageal Adenocarcinoma	NCT03995017

### Combination therapies of PARPis

Targeting PARP has recently been identified as a highly promising option for targeting GC. However, individual PARPis have limited efficacy. Combination strategies are desirable both to optimize the efficacy of PARPis and to expand the population of benefit (Fig. 3).

### Preclinical

Currently, PARPis are being studied in preclinical trials in synergy with a number of drugs in GC, several of which have been administered in combination therapy with encouraging results. Both as chemosensitizers or radiosensitizers, as well as in combination with other antineoplastic therapies. These preclinical trials provide a theoretical basis for the clinical application of synergistic regimens. In a preclinical study, the results demonstrated that combined treatment with PI3K and PARP inhibitors effectively inhibited the growth and migration of GC cells with ARID1A deficiency in vitro [94]. Moreover, because of the strong link between the efficacy of PARPis and impaired HR, a growing number of combination strategies with HR-deficient inducers have been developed for a variety of cancers [95–97]. In a recent study of GC, the study found that activation of c-MET increases tumor cell survival through the initiation of the DNA damage repair pathway. Herein, co-inhibition of c-MET and PARP enhances the levels of γ-H2AX and DNA damages in GC cells. The combinatorial treatment reduces the tumor and triggers apoptotic cell death growth in AGS xenograft models [98]. Furthermore, it is known from research that Checkpoint kinase 1 (Chk1) is a crucial regulator of cell cycle transition in DDR, it plays an important role in promoting the survival and growth of GC cells. Thus, combination with PARP1 inhibitor exhibited marked synergistic anticancer effect in both in vitro studies and in vivo experiments using a GC PDx model. Especially, Chk1 inhibitor combined with PARPis may be a more effective therapeutic strategy in GC. PARP inhibitor olaparib combined with WEE1/PLK1 dual inhibitor AZD1775 elicited potentiated anticancer activity through disrupting DDR signaling and the DNA damage checkpoint. AZD1775 abrogates olaparib-activated DNA damage checkpoint and causes mitotic DNA damage in GC cells. It sheds light on the combination strategy of WEE1/PLK1 dual inhibitors with PARPIs in the treatment of GC [81]. Based on these promising results, numerous clinical trials of combination strategies were conducted in GC patients. Table 3 summarizes these combinations of clinical trials that have been completed or are ongoing.

## Clinical

### PARPis and Chemoradiotherapy

#### Olaparib

PARPis were originally developed to enhance the antitumor activity of ionizing radiation and genotoxic agents [78, 99], they enhanced the efficacy of DNA alkylating agents, topoisomerase I poisons, and ionizing radiation [100]. Therefore, clinical trials (e.g. NCT01063517 and NCT01924533) have used this mechanism to show in more detail that this combination therapy shows a beneficial effect on patient survival. In a randomized, double-blind phase II clinical trial (NCT01063517), the combination of olaparib plus paclitaxel in patients with recurrent or metastatic GC with disease did not meet the primary endpoint of progression-free survival (overall population: hazard ratio [HR], 0.80; median PFS, 3.91 v 3.55 months, respectively; ATM low population: HR, 0.74; median PFS, 5.29 v 3.68 months, respectively). However, the combination of olaparib and paclitaxel was associated with a significant improvement in OAS versus paclitaxel plus placebo (13.1 vs. 8.3 months). This benefit was even more pronounced in patients with low ATM levels [101]. In the phase III GOLD trial (NCT01924533) clinical trial, olaparib/paclitaxel was generally well tolerated in both populations, however there was a trend in overall survival between the two populations but no statistical difference (median OS 8.8 months versus 6.9 months (HR = 0.79, *p* = .0262) [102]. Notably, ATM-negative patients treated with olaparib showed significantly improved remission rates compared to ATM-negative patients treated with paclitaxel alone (ORR 4.24, *p* = .0309), so PARPis may still be effective in selecting the correct biomarker for GC [103].

#### Veliparib

The results of A phase 1 dose-escalation study of veliparib with bimonthly FOLFIRI in patients with advanced solid tumors were also promising. Patients received veliparib (10–270 mg BID, days 1–5, 15–19) and FOLFIRI (days 1–3, 15–17) in three regimens containing 5-fluorouracil 2400 mg/m^2^: irinotecan 150 mg/m^2^ and folinic acid 400 mg/m^2^; irinotecan 180 mg/m^2^, folinic acid 400 mg/m^2^, and 5-fluorouracil 400 mg/m^2^ bolus, or irinotecan 180 mg/m^2^. Besides, ORR was 17.6% [104]. The acceptable safety profile and preliminary antitumor activity of veliparib plus FOLFIRI support further evaluation of this combination.

### PARPis and targeted drugs

Anti-angiogenic therapies are known to induce a hypoxic cellular state that leads to downregulation of HR repair genes (BRCA1, BRCA2, and RAD51, among others), thereby enhancing PARPis sensitivity [105]. Furthermore, the studying of Olaparib and Ramucirumab in Treating Patients With Metastatic or Locally Recurrent Gastric or Gastroesophageal Junction Cancer That Cannot Be Removed by Surgery (NCT03008278) is ongoing. Of these, ramucirumab is a humanized monoclonal antibody that specifically blocks VEGFR2 and downstream angiogenesis-related pathways. And FDA has approved ramucirumab for the treatment of advanced gastric cancer or gastroesophageal union adenocarcinoma. AZD6738 is an orally active, selective ATR kinase inhibitor. The combination of AZD6738 and olaparib has received preclinical validation. A Phase I/II clinical study of ceralasertib in combination with olaparib (NCT02264678) is also currently underway and is highly anticipated.

### PARPis and immune checkpoint inhibitors

In a Phase 1a / b clinical trial of pamiparib in combination with anti-PD-1tislelizumab in patients with advanced solid tumors (NCT02660034), 49 patients were enrolled (median age 63 years [IQR 55–67]), all of whom received at least one dose of pamiparib or tiselzumab. At a median follow-up of 8·3 months (IQR 4·8–12·8), ten (20%) of 49 patients achieved an objective response according to Response Evaluation Criteria in Solid Tumors (RECIST) version 1.1, including two complete responses and eight partial responses. And no grade 5 adverse events were reported. Pamiparib with tislelizumab was generally well tolerated and associated with antitumor responses and clinical benefit in patients with advanced solid tumors supporting further investigation of the combination of pamiparib with tislelizumab [106]. Equally, the phase 2 MEDIOLA basket trial assessed the efficacy and safety of chemo-free combination of olaparib and durvalumab in patients with solid tumors, including ovarian cancer, breast cancer and GC (NCT02734004). Combination of olaparib and durvalumab showed promising antitumor activity and safety similar to that previously observed in olaparib and durvalumab monotherapy studies. In GC, there are increasing clinical trials (NCT02264678, NCT04178460) underway that will help decipher the exact role of PARPis in combination with the anti-PD1 / PD-L1 strategy (Table 3).

## Conclusions

Over the past few years, we have witnessed the use of PARPis in the treatment of GC and see a bright future in various preclinical and clinical data. However, because GC are highly heterogeneous tumors, PARPis may still be more effective in GC with specific biomarkers, such as the use of ATM loss as a predictive biomarker of GC response. FDA approval of Lynparza was accompanied by approval of a genetic test called BRACAnalysis CDx to detect the presence of BRCA gene mutations (gBRCAm) in blood samples from ovarian cancer patients [107]. We also expect that appropriate genetic assays will be available to monitor the reliability of PARPis for use in GC. A better understanding of PARP inhibitor resistance and the underlying mechanisms involved in its mechanism of action will also help in the development of new therapeutic strategies to address this issue [108]. Importantly, there is a need for novel adaptive trial designs to validate the various mechanistic hypotheses emerging in GC. By addressing these gaps in our knowledge, the use of PARPis in GC treatment will continue to expand as a “beacon of hope” in our anticancer armamentarium.

## Data Availability

Not applicable.

## Abbreviations

DDR: DNA damage response
PARP: Poly (ADP-ribose) polymerase
PARylation: Poly (ADP-ribosylation)
DSB: DNA single strand breaks
HR: Homologous recombination
GC: Gastric cancer
PARPis: PARP inhibitors
SSB: Single-strand breaks
HRR: Homologous recombination repair
HRD: HRR deficiency
PTM: Post-translational modification
NAD+: Nicotinamide adenine dinucleotide
PARG: PAR glycolytic enzymes
ARH3: ADP-ribosyl hydrolase 3
ARTDs: Diphtheria toxin-like ADP-ribosyltransferase
cNHEJ: Classical non-homologous end joining
CCCH: Cys-Cys-Cys-His zinc finger
AIF: Apoptosis-inducing factor
BER: Base excision repair
MMEJ: Microhomology-mediated end joining
POLQ: Polymerase
STING: Stimulator of IFN genes
TIL: Tumor-infiltrating lymphocytes
FDA: The Food and Drug Administration
EMA: European Medicine Agency
VEGF: Vascular endothelial growth factor
SDC-4: Transmembrane signaling protein syndecan-4
HIF: Hypoxia inducible factor
DNMT1: DNA (cytosine-5-)-methyltransferase 1
FOXO: Forkhead box O
ATM: Ataxia telangiectasia mutant
BD: Orally twice daily
RECIST: Response Evaluation Criteria in Solid Tumors
gBRCAm: BRCA gene mutations
